# Interventions for Neural Plasticity in Stroke Recovery

**DOI:** 10.26502/aimr.0217

**Published:** 2025-08-25

**Authors:** Jaylan Patel, Iris Shim, Devendra K. Agrawal

**Affiliations:** 1 Department of Translational Research, College of Osteopathic Medicine of the Pacific, Western University of Health Sciences, Pomona, California 91766 USA.

**Keywords:** Brain-derived neurotrophic factor, Cognitive behavioral therapy, Nerve growth factor, Neuroimaging, Neuroplasticity, Physical therapy, Rehabilitation, Stroke, Stroke interventions

## Abstract

Stroke is a leading cause of long-term disability, and enhancing neural plasticity is a central strategy in promoting functional recovery. This review examines a range of interventions that target plasticity to improve outcomes in stroke survivors. Neural plasticity is assessed using neuroimaging tools, such as fMRI, EEG, and fNIRS, as well as clinical scales, including the Fugl-Meyer Assessment (FMA) and the Modified Rankin Scale (mRS). Biomarkers, like brain-derived neurotrophic factor (BDNF), GABA, and nerve growth factor (NGF), are also useful for predicting patient outcomes. These tools offer insight into recovery potential and intervention effectiveness. The interventions discussed include physical therapy, cognitive behavioral therapy (CBT), dietary support, and emerging technologies such as virtual reality, video games, and exoskeleton-assisted training. Pharmacological strategies, including Levodopa, selective serotonin reuptake inhibitors (SSRIs), and ginkgo diterpene lactone meglumine (GDLM), have shown mixed results, while stem cell therapies remain under investigation. Physical therapy remains the foundational treatment, but other interventions may provide added benefit depending on patient characteristics. This review highlights the need for a personalized, multidimensional approach to stroke rehabilitation. Continued research is necessary to refine these therapies and optimize recovery through tailored treatment strategies.

## Introduction

Stroke is a leading cause of long-term disability and mortality worldwide, affecting nearly 12 million individuals annually and accounting for approximately 7 million deaths each year [1]. Stroke is clinically defined as a sudden neurological deficit resulting from an acute focal injury to the central nervous system (CNS)—including the brain, retina, or spinal cord—caused by a vascular event [2]. The vast majority of strokes are ischemic, typically caused by arterial occlusion, but a less common subtype of ischemic strokes, venous infarction, results from cerebral venous sinus thrombosis. Hemorrhagic strokes, comprising 10–40% of cases depending on regional variation, occur due to rupture of cerebral vessels and include both intracerebral and subarachnoid hemorrhages [3]. While some patients experience transient symptoms, imaging studies reveal that many cases previously classified as transient ischemic attacks (TIA) involve actual infarction, putting these patients at high risk for recurrence [2]. Despite advances in acute stroke management, many survivors experience persistent deficits due to irreversible neuronal loss and limited capacity for spontaneous neural regeneration [4].

A key determinant of post-stroke recovery is the brain’s ability to undergo neural plasticity—a dynamic process by which the CNS reorganizes its structure and function in response to injury [5]. These neuroplastic adaptations underlie the potential for functional restoration and are targeted by most therapeutic interventions aiming to improve outcomes after stroke. However, the extent and pattern of post-injury plasticity can vary widely, and optimizing adaptive reorganization while mitigating maladaptive responses remains a persistent challenge in stroke rehabilitation research.

Plasticity manifests through diverse and interacting mechanisms, including cortical remapping, axonal sprouting, dendritic arborization, and synaptic reorganization [5–10]. These processes are modulated by both intrinsic biological factors, such as the severity and location of injury, patient age, molecular mediators, and extrinsic influences, such as rehabilitative training and neuromodulatory interventions. Notably, a temporally limited window of heightened plastic potential following stroke has been identified, suggesting that early and targeted intervention may be critical for optimizing recovery [9]. Given the centrality of neuroplasticity in post-stroke rehabilitation, there is growing interest in identifying and optimizing interventions that enhance adaptive plasticity while minimizing maladaptive changes [10].

In this review article, we critically discussed the following points: (i) the mechanisms through which the brain attempts to recover function via neural plasticity, (ii) evaluation and comparison of current pharmacological, behavioral, neuromodulatory, and technological strategies designed to enhance post-stroke neuroplasticity, and (iii) synthesis of clinical and experimental evidence to assess the efficacy, limitations, and future potential of these interventions in promoting long-term recovery.

## Pathophysiology of Stroke and Neural Plasticity

### Pathophysiology of Stroke

The pathophysiology of ischemic stroke involves a cascade of deleterious events. Interruption of cerebral blood flow initiates rapid energy failure, disrupting ionic gradients and leading to calcium influx, glutamate-mediated excitotoxicity, oxidative stress, mitochondrial dysfunction, and ultimately necrosis and apoptosis [11–13]. These mechanisms cause irreversible damage not only to neurons but also to glial and vascular endothelial cells, amplifying neuroinflammation and tissue injury [11–13]. This complexity renders spontaneous functional recovery challenging, particularly in the absence of targeted intervention [13].

### Mechanistic Distinctions Between Ischemic and Hemorrhagic Strokes

**Figure 1:**
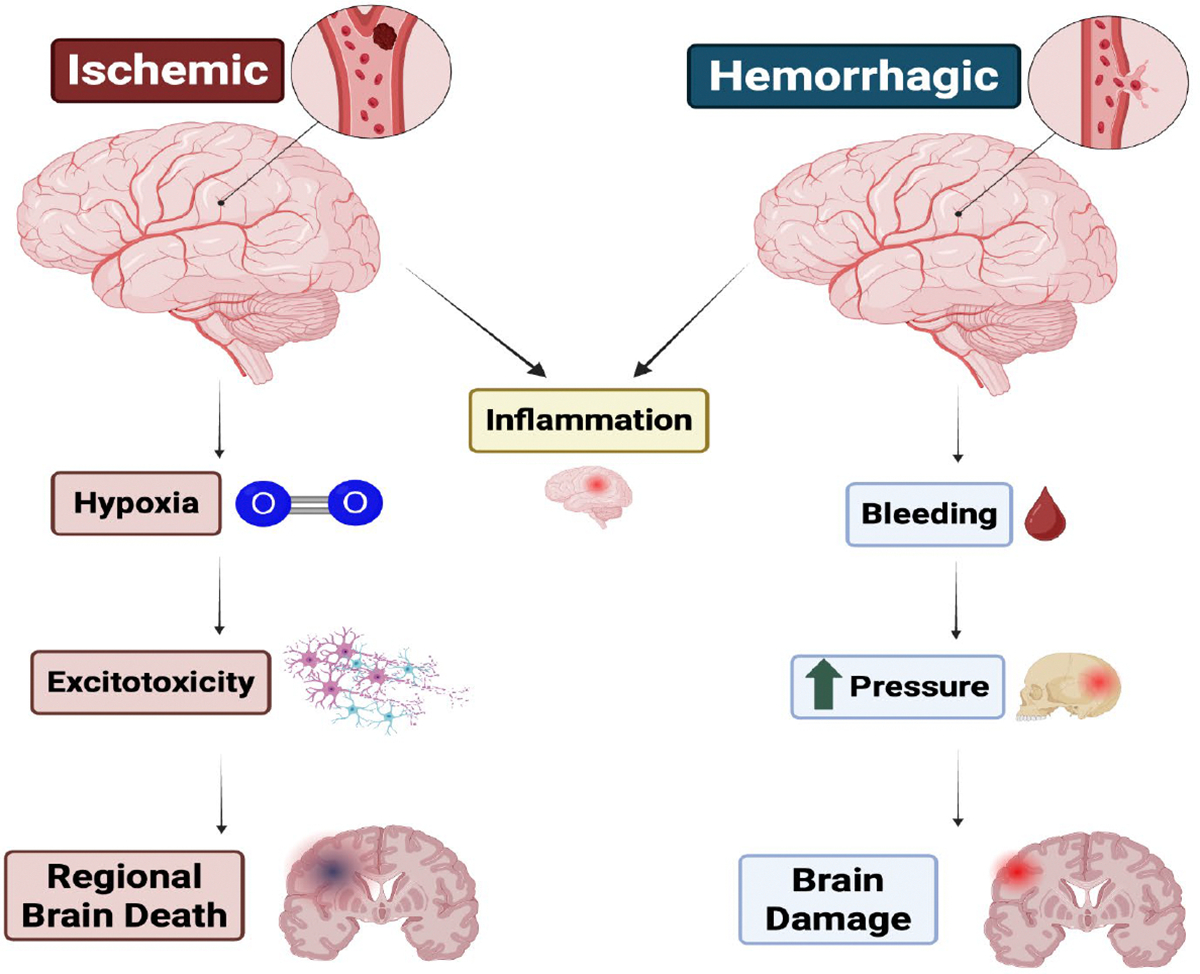
Schematic diagram comparing ischemic and hemorrhagic strokes and their mechanisms.

Although ischemic and hemorrhagic strokes share common clinical features, their pathophysiological mechanisms diverge significantly, influencing the course of recovery. Ischemic stroke results from impaired blood flow, leading to hypoxia, metabolic failure, excitotoxicity, and inflammatory cascades. Hemorrhagic stroke, in contrast, causes direct mechanical damage from hematoma expansion and neurotoxic effects of blood products, such as hemoglobin breakdown and iron deposition [14]. A comparison between the two types of strokes is illustrated in Figure 1. The scope for neuroplastic recovery may differ between subtypes, with ischemic stroke more extensively studied in relation to post-injury reorganization and rehabilitative responsiveness [11–13].

### Neural Damage

At the cellular level, ischemia-induced energy failure disrupts ATP-dependent ion transport mechanisms, causing depolarization and intracellular calcium overload [11]. This initiates a pathological cascade leading to intracellular calcium accumulation, release of excitatory neurotransmitters such as glutamate, generation of reactive oxygen species (ROS), and activation of pro-apoptotic signaling pathways [11–13]. Inflammatory mediators and blood-brain barrier breakdown further exacerbate injury to neurons, astrocytes, oligodendrocytes, and endothelial cells, contributing to a dynamic and self-reinforcing cycle of cell death [12–13]. These combined effects contribute to long-term disability and highlight the brain’s limited ability to regenerate after stroke [4].

### Role of Neural Plasticity in Stroke Recovery

**Figure 2:**
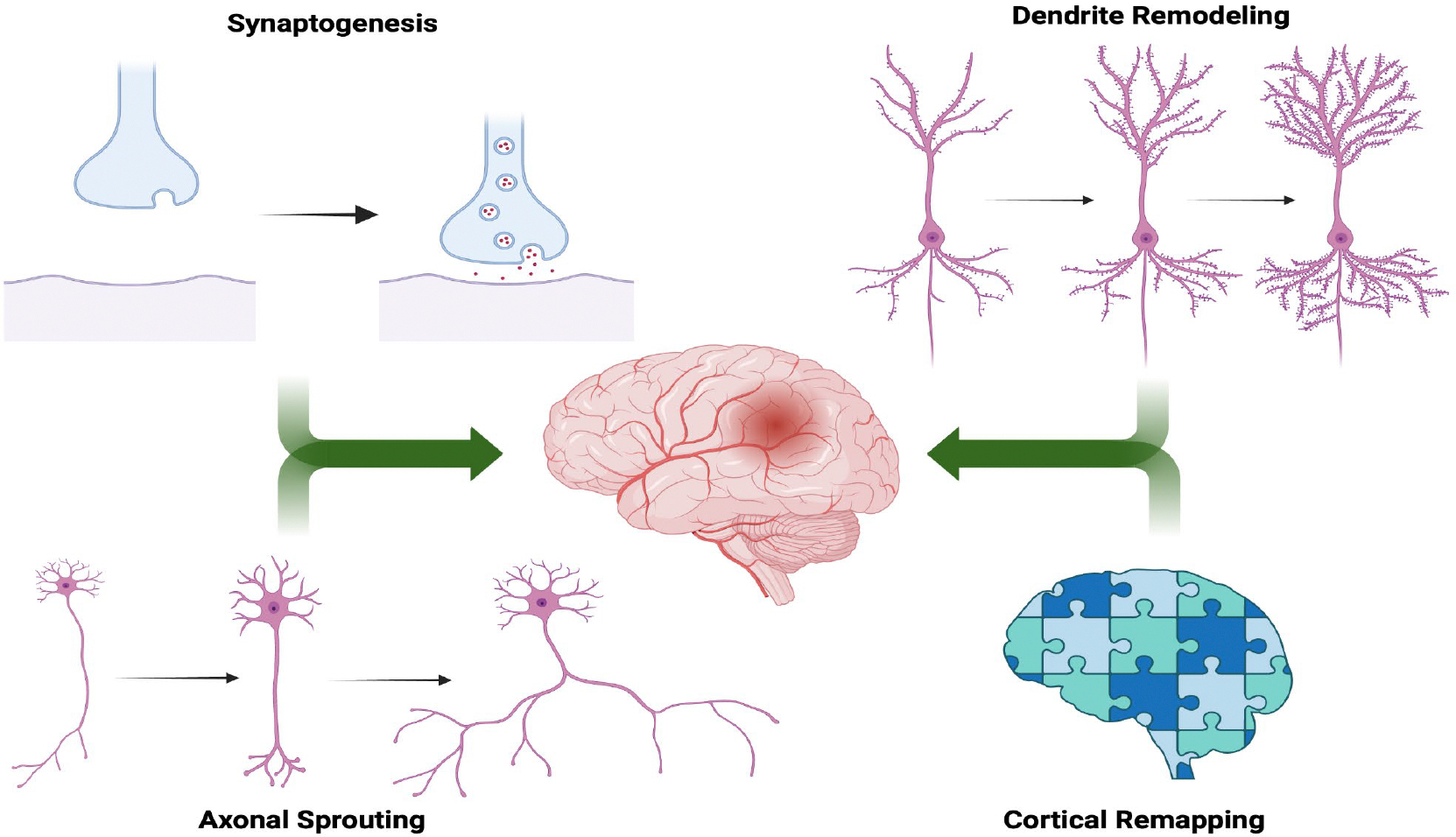
Schematic diagram showing the major mechanisms of neural plasticity, which allow the brain to recover after a stroke.

In the aftermath of a stroke, the brain engages compensatory mechanisms that attempt to restore lost function by reorganizing surviving neural networks [5]. These neuroplastic processes are most active during the subacute phase post-injury, a critical window during which rehabilitation has the greatest potential to improve outcomes [9]. Multiple mechanisms underlie the brain’s capacity to reorganize following stroke, each contributing to functional recovery through network remodeling. Cortical Remapping is the shifting of the functional representations of motor and sensory systems to perilesional or contralesional regions following injury. This redistribution, particularly in the primary motor and premotor cortices, has been demonstrated in both animal models and human neuroimaging studies [7]. Axonal Sprouting occurs when the surviving neurons generate new axonal projections to replace connections lost due to infarction. These sprouts often target denervated areas, supporting the restoration of disrupted circuits and contributing to regained motor and sensory functions [5–6]. Recovery is also facilitated by synaptogenesis, or increased synapse formation, and dendritic remodeling, which enhance synaptic density and signal propagation within reorganized neural pathways [5–8]. These mechanisms are key components to post-stroke recovery as illustrated in Figure 2. However, not all forms of plasticity are beneficial. For instance, post-stroke studies have shown that in some patients, excessive interhemispheric inhibition from the contralesional motor cortex suppresses activity in the ipsilesional hemisphere during voluntary movement, correlating with poorer motor outcomes [15]. Therefore, a major therapeutic goal is to promote adaptive plasticity while minimizing processes that interfere with recovery [10].

## Assessment and Indicators of Neural Plasticity

### Neuroimaging and Cognitive Assessment Tools

Understanding neural plasticity is crucial for improving stroke rehabilitation and optimizing recovery strategies. Imaging plays a vital role in tracking and quantifying neural plasticity (Table 1). Functional magnetic resonance imaging (fMRI) is one of the most widely used techniques for assessing brain function. It provides high spatial resolution, enabling the detection of changes in blood supply and the evaluation of neuronal networks [16–17]. fMRI can measure functional adaptations in response to various conditions, such as deafness [18]. In stroke, fMRI helps identify changes in connectivity within specific brain regions, reflecting plasticity [19], and can also assess cerebral blood flow in the acute phase of stroke [20]. Another widely used imaging modality is electroencephalography (EEG), which records the brain’s electrical activity and offers an indirect measure of neuronal function [21–22]. Unlike fMRI, EEG provides excellent temporal resolution, allowing real-time observation of neural events [23]. This temporal precision gives EEG an advantage when evaluating the timing of brain activity. EEG has been employed to assess changes in brain plasticity following therapeutic interventions [24] and brain injuries [25–26]. fMRI and EEG remain the most used imaging techniques in clinical research for evaluating the spatial and temporal aspects of neural plasticity following stroke. A newer imaging modality, functional near-infrared spectroscopy (fNIRS), provides a non-invasive method for studying cognitive function in conjunction with brain activity [27–28]. It does this by measuring changes in oxygenated and deoxygenated hemoglobin concentrations during neural activation [29]. fNIRS has been used to monitor neuroplastic changes following stroke and to identify shifts in cortical function associated with recovery [30]. Collectively, these tools provide valuable insights into the brain’s capacity for reorganization and are essential for evaluating the effectiveness of interventions aimed at enhancing neural plasticity during stroke rehabilitation.

There are several clinical scales commonly used to assess functional outcomes following a stroke (Table 1). The Fugl-Meyer Assessment (FMA) is a widely utilized tool designed to quantify motor impairment in stroke patients [31]. It has been validated as a reliable and sensitive measure for evaluating both upper and lower extremity function [32]. Therefore, it is particularly useful in rehabilitation research focused on motor recovery. Another frequently used measure is the modified Rankin Scale (mRS), which assesses the degree of overall disability or dependence in daily activities after a stroke. However, studies have shown that while the mRS is useful for broad clinical assessments, its consistency and interrater reliability are modest [33–34]. These scales are frequently used as outcome measures in clinical trials and therapeutic evaluations, so understanding their respective strengths and limitations is essential when interpreting the efficacy of post-stroke interventions.

### Genetic and Neurochemical Biomarkers

Recent research has shown that specific biomarkers are associated with poorer outcomes after stroke and reduced therapeutic efficacy. One such biomarker is brain-derived neurotrophic factor (BDNF), a gene in which common polymorphisms have been linked to differences in post-stroke recovery [35]. Studies have demonstrated that this polymorphism is associated with decreased aphasia severity and greater responsiveness to aphasia treatment [36–37]. Furthermore, the effectiveness and variability of interventions aimed at promoting neural plasticity appear to be influenced by a patient’s BDNF genotype [38–39]. Identifying genetic markers associated with differential treatment responses can guide the selection of therapeutic strategies most likely to be effective across diverse patient populations. Additionally, screening for such polymorphisms may help clinicians personalize rehabilitation plans, improving outcomes by tailoring interventions to the patient’s genetic profile.

In addition to genetic factors, neurochemical modulators such as γ-aminobutyric acid (GABA) also influence stroke recovery and neural plasticity. Changes in GABA receptor availability have been linked to motor improvement following ischemic stroke [40]. While GABA normally helps regulate brain activity, elevated levels after stroke have been associated with impaired memory and reduced synaptic plasticity [41]. One proposed mechanism is interhemispheric inhibition, where GABA signaling from the unaffected hemisphere suppresses activity in the affected one, limiting recovery. Additionally, animal models that recovered from stroke showed a loss of this GABA-mediated inhibition, suggesting it may be necessary to reduce inhibition to support plasticity and functional improvement [42]. A GABA antagonist has been proposed as a potential therapeutic strategy to reduce excessive inhibition and promote recovery. However, clinical studies using a GABA α5 receptor antagonist have not demonstrated significant improvements in functional outcomes [43]. While targeting GABAergic signaling remains a promising area of research, current evidence is limited. More studies are needed to better understand the role of tonic inhibition in post-stroke plasticity and to determine whether modulating this pathway can yield meaningful clinical benefits. Nerve growth factor (NGF) is another biomarker associated with functional recovery following stroke. NGF plays a critical role in the growth, maintenance, and survival of afferent neurons [44], making it a key factor in post-stroke neural repair. Elevated serum NGF levels after acute ischemic stroke have been significantly correlated with more favorable functional outcomes [45]. As such, promoting NGF expression may not only enhance neural plasticity but also serve as a predictive marker of recovery. While early studies suggest potential therapeutic applications for NGF, current research remains preliminary, and its clinical use is not yet established [46–47]. Tracking biomarkers, like NGF and GABA, is essential for quantifying intervention efficacy as more become available.

## Interventions and Management of Neural Plasticity following Stroke

### Rehabilitation, Psychotherapy, and Nutrition

Rehabilitation is a major component of post-stroke care, not only for restoring motor function but also for promoting neuroplasticity. Among rehabilitative strategies, physical therapy has been extensively studied for its ability to drive cortical reorganization and functional recovery. Aerobic and task-specific exercises, such as treadmill training, have been shown to enhance walking speed and endurance [48–49], with some evidence suggesting overground walking may be even more effective [50]. Upper extremity interventions, including constraint-induced movement therapy, target learned non-use and promote re-engagement of affected cortical areas. However, suboptimal use often limits its efficacy [51]. When appropriately administered, these therapies contribute to activity-dependent plasticity, strengthening neural circuits involved in motor control. Therefore, physical exercise aids in motor recovery by promoting widespread neuroplastic changes. This direct link between physical activity and neural plasticity is evident as exercise has been shown to upregulate neurotrophic factors, such as BDNF, and enhance hippocampal neurogenesis [52–54]. Long-term running increases BDNF expression, a key mediator of synaptic plasticity and neuronal survival, which may facilitate cognitive recovery after stroke [52]. Additionally, early-life or post-stroke treadmill exercise has been linked to improved hippocampal neuroplasticity and structural remodeling [53–54], suggesting a lasting influence on brain repair mechanisms. These findings underscore the role of exercise as a potent, non-invasive intervention for modulating neuroplasticity during stroke recovery.

Given the high prevalence of post-stroke depression and anxiety, addressing emotional and cognitive outcomes is essential for comprehensive recovery. Cognitive behavioral therapy (CBT) has demonstrated clinical efficacy in improving mood symptoms in stroke survivors [55]. Beyond symptom management, CBT appears to facilitate neuroplastic changes by modifying structural plasticity within the amygdala [56] and altering functional connectivity across emotion-regulation networks [57]. When combined with physical therapy, CBT has also been shown to enhance cognitive function through mechanisms of cortical reorganization. In one study, patients receiving both CBT and physical therapy exhibited significantly greater EEG-based markers of cortical reorganization, compared to those undergoing physical therapy alone [58]. These findings suggest that CBT may augment neural recovery by promoting functional brain remodeling, further supporting its role as a valuable tool in post-stroke rehabilitation.

Dietary factors also influence neural plasticity and stroke outcomes. Deficiencies in key micronutrients have been associated with worse neurological recovery. For example, vitamin B12 deficiency has been linked to decreased motor function, greater infarct volume, and altered mitochondrial metabolism in animal models of ischemic stroke [59–60]. Prenatal folate deficiency impairs neurodevelopment and leads to worse stroke outcomes in offspring [61], while low vitamin D levels are correlated with increased stroke severity in humans [62]. Although the effects of post-stroke dietary supplementation remain under investigation, these findings highlight the role of diet in shaping the brain’s capacity for plastic change and recovery. Therefore, it can be assumed that a sufficient diet is another key component for promoting neural plasticity in stroke recovery.

### Non-Invasive Brain Stimulation

Non-invasive brain stimulation is another promising tool used to enhance neural plasticity following stroke. Transcranial magnetic stimulation (TMS) applies targeted magnetic pulses to modulate cortical excitability and has been studied in both psychiatric and neurological conditions [63]. Repetitive TMS protocols are designed to either inhibit or excite targeted brain regions depending on the frequency and location of stimulation. However, the effectiveness of rTMS in stroke recovery appears to depend heavily on the stimulation site and patient characteristics. For example, one study found that low-frequency inhibitory stimulation over the contralesional motor cortex did not result in significant improvements in motor recovery compared to control in patients with subacute ischemic stroke [64], highlighting the importance of stimulation site selection. In another study, both excitatory and inhibitory TMS protocols were evaluated in acute stroke patients. High-frequency stimulation was applied to the ipsilesional motor cortex, while low-frequency inhibitory pulses were delivered to the contralesional side. The treatment group showed significantly greater motor recovery and decreased GABA levels in the ipsilesional cortex [65]. This further emphasizes the link between reduced GABA-mediated inhibition and improved plasticity. Similarly, inhibitory rTMS over the unaffected hemisphere led to better upper limb recovery than facilitative protocols, specifically in patients with high corticospinal tract integrity [66]. These findings support the idea that targeting the GABAergic inhibitory pathway may enhance the brain’s capacity for reorganization. Further research has explored combining TMS with other neuromodulatory techniques. Other studies showed that pairing TMS with transcranial direct current stimulation produced significantly greater improvements in motor function than TMS alone [67–68]. These multimodal approaches may work synergistically to promote plasticity through multiple pathways. TMS represents a promising, noninvasive strategy to modify brain networks involved in recovery directly. While results are encouraging, further research is needed to optimize stimulation protocols, identify ideal patient populations, and fully understand the neurobiological mechanisms that underlie post-stroke recovery.

### Pharmacology

Pharmacological interventions are another active area of investigation aimed at enhancing neural plasticity after stroke. Levodopa (L-Dopa), a dopamine precursor traditionally used in the treatment of Parkinson’s disease, is one possible intervention under investigation. Older studies suggested that Levodopa administration significantly improves motor performance, both as a standalone treatment [69] and when combined with physical therapy [70]. More recent research has begun to explore the underlying mechanisms by which Levodopa may promote plasticity. For example, Levodopa has been shown to upregulate oligodendrocyte precursor cells, supporting myelination and repair [71], and to modulate peripheral immune responses, potentially reducing secondary damage and enhancing neuroprotection [72]. These findings suggest that Levodopa may enhance neural recovery through both central and peripheral mechanisms. However, its clinical application in diverse stroke populations remains uncertain. Much of the current evidence supporting Levodopa’s efficacy comes from older or small-scale studies. More rigorous, large-scale trials are needed to validate its therapeutic potential, particularly when used in combination with other interventions.

Another pharmacologic approach under investigation involves selective serotonin reuptake inhibitors (SSRIs). Widely prescribed for depression, SSRIs have also been linked to enhanced neural plasticity and improved learning in non-stroke populations [73]. Despite this potential, clinical trials assessing the role of SSRIs in post-stroke recovery, particularly fluoxetine, have reported no significant improvement in functional outcomes compared to placebo [74–76]. As a result, current evidence does not strongly support SSRIs as an effective standalone intervention for enhancing motor recovery after stroke. Further research may help clarify whether specific patient subgroups or combination therapies could benefit from SSRI use.

A unique pharmacological intervention more commonly used in China is ginkgo diterpene lactone meglumine (GDLM). GDLM is primarily administered after an acute ischemic stroke and is thought to exert neuroprotective effects that support functional recovery. Clinical studies have demonstrated improvements in both cognitive function [77] and disability scores, as measured by the modified Rankin Scale (mRS) [78], following GDLM treatment. These findings suggest that GDLM may help promote both cognitive and motor recovery when compared to no intervention. Additionally, GDLM has shown beneficial effects when used in combination with other therapies. For example, when administered alongside aspirin, GDLM’s antiplatelet properties were associated with improved post-stroke prognosis compared to aspirin alone [79]. It has also been studied in conjunction with repetitive transcranial magnetic stimulation, where co-administration resulted in enhanced cognitive and neurological recovery [80]. However, as this study lacked a TMS-only control group, the specific contribution of GDLM remains uncertain. Nonetheless, GDLM appears to be a promising agent that may facilitate neuroplasticity-driven recovery, supporting both motor and cognitive outcomes after stroke.

### Stem Cell Therapy

Given the high prevalence of stroke and the current lack of standardized interventions to reliably enhance neural plasticity, several novel therapies are under investigation. Stem cell therapy is one such approach that aims to promote brain repair and plasticity by introducing stem cells into damaged neural tissue. Although this strategy has been widely studied in preclinical and early clinical settings, results have been mixed. Trials involving intra-arterial delivery of bone marrow mononuclear cells and autologous modified mesenchymal stem cells have demonstrated safety in stroke patients but failed to show significant improvements in modified Rankin Scale (mRS) scores at three months post-treatment [81–82]. Another trial using a bone marrow-derived, allogeneic multipotent adult progenitor cell product administered within 18 to 36 hours of stroke onset similarly reported no significant short-term benefit on functional outcomes [83].

In contrast, other studies have found that mesenchymal stem cell therapy can enhance motor performance and increase activity in the motor cortex compared to controls [84–85]. These trials used the Fugl-Meyer Assessment and reported significant improvements in treated patients. Additionally, even in the study that observed gains in FMA scores, mRS outcomes remained unchanged. This highlights a potential disconnect between disability measures and motor-specific functional recovery. These findings suggest that while stem cell therapy shows promise in enhancing motor function and neural reorganization, its clinical utility may depend on patient selection, timing of administration, and the outcome measures used. The FMA may offer greater sensitivity in detecting motor recovery compared to the mRS, which captures broader functional disability. Future research should focus on refining protocols, identifying responsive patient populations, and standardizing the most relevant outcome to better evaluate the therapeutic potential of stem cell–based interventions.

### Technology-Based Interventions

Today, many emerging stroke interventions utilize technology to enhance neural plasticity and functional recovery in post-stroke care. One such approach is exoskeleton-mediated physical therapy, which uses wearable robotic devices to assist with repetitive, task-specific training. Studies have demonstrated that this method can improve motor independence and clinical outcomes in stroke survivors [86–87]. While current evidence suggests that exoskeleton-assisted therapy is comparable in effectiveness to conventional physical therapy [88–89], the ability to deliver high-repetition, consistent training may offer added benefits. Repetitive movement is known to drive activity-dependent plasticity, and more frequent engagement of motor circuits could promote neuroplastic changes, especially when used in home-based settings.

Similarly, virtual reality (VR) is being explored as a means to promote cognitive recovery through immersive and adaptive environments. VR-based rehabilitation has been shown to improve depressive mood, attention, and spatial awareness in post-stroke patients [90]. When compared to traditional or adaptive pen-and-paper tasks, VR training resulted in greater cognitive gains and better retention over time [91]. These findings suggest that VR may facilitate more effective plasticity in cognitive domains by engaging users in dynamic, individualized training experiences that support the brain’s natural capacity to rewire after injury. Video game-based rehabilitation is a related intervention that focuses primarily on motor recovery rather than cognitive enhancement. Studies have demonstrated that video game-based therapy can significantly improve motor function in stroke patients [92–93], with outcomes comparable to those of conventional in-clinic physical therapy [93]. This suggests that video game rehabilitation may serve as a more accessible and cost-effective alternative for some patients, especially for those pursuing home-based recovery. Furthermore, games designed to integrate cognitive challenges alongside motor tasks may offer dual benefits, potentially enhancing both cognitive and physical recovery. However, further research is needed to define optimal protocols and delivery platforms to determine the most effective approach for both virtual reality and video game-based interventions.

### Long-term effects

Long‑term recovery after stroke depends on sustaining adaptive neuroplasticity beyond the subacute “sensitive period,” when plasticity transitions to a lower‑gain, experience‑dependent state that typically requires ongoing behavioral dosing, neuromodulation “boosters,” or targeted pharmacologic augmentation to retain gains[78–79, 94–95]. Irreversible tissue loss, persistent network inhibition (e.g., excess GABAergic tone, abnormal interhemispheric inhibition), and limited intrinsic regenerative capacity continue to constrain spontaneous recovery over time [22, 77, 80, 95–96]. In practice, task‑specific training is the most consistently beneficial approach, but its effects attenuate without maintenance; even where the biological rationale is strong (e.g., exercise‑induced BDNF upregulation), high‑quality ≥6–12‑month data remain limited [48–49, 52–54, 97–100].

Across neuromodulation (rTMS/tDCS), pharmacology (levodopa, SSRIs, GABAA_AA α5 antagonists), GDLM, stem cells, and exoskeletons/VR, the same pattern repeats: short‑term gains are common; durable effects are inconsistent, moderator‑dependent, and often measurement‑sensitive [43, 64–68 74–76, 81–93, 96, 101–105, 106–120]. Fugl‑Meyer (FMA) often detects persistent motor improvements when global disability scales (mRS) do not, creating the illusion of “lost effects” [31–34]. Pragmatically, booster paradigms, biomarker‑guided personalization (e.g., BDNF genotype, GABA PET/MRS), and scalable home‑based digital dosing (VR/robotics) are the most plausible strategies to convert early gains into durable, clinically meaningful outcomes [35–42, 77, 79, 86–93, 104, 113–120].

## Conclusion

Enhancing neural plasticity remains a central goal in optimizing stroke recovery. While no single intervention benefits all patients equally, physical therapy remains the cornerstone of rehabilitation. Despite this, each patient will often benefit from alternative modalities or personalized treatment plans that may offer additional benefits. As the field progresses, future research should focus on refining these interventions and clarifying how they influence recovery, both independently and in combination, to improve patient outcomes.

## Figures and Tables

**Table 1:** Summary of the Imaging and Clinical Tools for Assessing Neural Plasticity.

Assessment Tool	Clinical/Functional Parameter	Clinical Utility of the Findings
Functional Magnetic Resonance Imaging (fMRI)	• Blood flow changes	• Maps brain connectivity and activity • Spatial resolution
Electroencephalography (EEG)	• Electrical brain activity	• Detects real-time brain responses • Temporal resolution
Near-infrared spectroscopy (fNIRS)	• Hemodynamic changes	• Cognitive and brain function assessment
Fugl-Meyer Assessment	• Motor impairment after a stroke	• Useful for interventions targeting motor function
Modified Rankin Scale	• Overall disability after a stroke	• Broad functional outcomes • Modest reliability

**Table 2:** Summary of the Therapeutic Interventions to Enhance Neural Plasticity After Stroke

Intervention	Mechanism(s)	Key Points	Long Term Effects
Physical Therapy	↑ BDNF Cortical reorganization Task-specific repetition	Foundational intervention Enhances neuroplasticity with consistent application Exoskeleton-assisted may improve patient access and consistency	Benefits persist only with continued/intensive or home-based dosing Attenuation when intensity drops Biological plausibility is strong, but ≥12‑month data are limited
Cognitive Behavioral Therapy	Amygdala plasticity Emotion regulation	Dual benefit for mood and cognition Complements physical therapy	Sustained mood benefits are plausible Durable motor effects are unproven
Diet	Overall health and well-being	Prevents worsening of stroke outcomes Indirectly supports plasticity	Deficiency correction helps, but supplementation alone lacks evidence for durable motor gains
Transcranial Magnetic Stimulation	Cortical excitability ↓ GABA inhibition	Modulates activity in affected networks Effect depends on protocol No standardized booster	Early gains common Long‑term durability is inconsistent and protocol/phenotype dependent paradigms CST integrity and cortical inhibition state moderate persistence
Levodopa	Dopaminergic modulation	May enhance motor recovery through neuroplasticity and immune support	Short‑term gains (often when paired with PT); no convincing ≥6–12‑month superiority
Ginkgo Diterpene Lactone Meglumine	Neuroprotection Antiplatelet	Shows promise in studies from China May enhance the effect of other therapies	Short‑term cognitive and mRS gains Insufficient long-term data and unclear additive effect when combined with other modalities (e.g., rTMS)
Stem Cell Therapy	Cell replacement Brain repair	May aid motor recovery Further validation and scaling needed FMA gains can out persist, but mRS often unchanged	True durable, generalizable effects not yet shown in phase 2/3 trials (e.g., TREASURE)
Virtual Reality	Multisensory feedback Real-time adaptation	Engages attention and spatial networks Enhances cortical activity	Improves retention of cognitive and motor skills, potentially better engagement for prolonged training Long-term RCT evidence is still scarce
Video Game Therapy	Repetition Dual-task learning	Combines motor and cognitive rehab in an accessible format	Durable effects suggested but underpowered long-term RCTs

## Key Points and Outstanding Questions

- Physical therapy continues to be the most reliable intervention for promoting neural plasticity and functional recovery post-stroke.
- Biomarkers, such as BDNF, GABA, and NGF, could help predict recovery potential and personalize treatment, but their clinical utility remains limited by variability in measurement and interpretation.
- Combining interventions may yield synergistic effects, but optimal pairings are still unclear.
- TMS shows promise, but challenges remain in determining ideal stimulation parameters, cortical targets, and timing across stroke phases.
- There is inconsistency in outcome measures, such as mRS vs. FMA, complicating the evaluation of motor-specific recovery versus global disability.
- Pharmacologic agents, like Levodopa and GDLM, show early promise but require larger, high-quality trials to confirm efficacy and safety.
- Stem cell therapies are safe and may enhance motor plasticity, but clinical benefits remain modest and dependent on cell type, delivery method, and timing.
- Virtual reality and video game–based therapies are accessible and engaging but lack standardization in protocols, dosing, and target populations.
- What is the best way to combine neuroimaging, clinical scales, and biomarkers to assess plasticity in a clinically meaningful way?
- How do patient-specific factors (e.g., stroke subtype, lesion location, comorbidities) influence responsiveness to various interventions?
